# Exploring the Role of Fibrin Gels in Enhancing Cell Migration for Vasculature Formation

**DOI:** 10.3390/jfb15090265

**Published:** 2024-09-12

**Authors:** Joana A. Moura, Hugh J. Barlow, Shareen H. Doak, Karl Hawkins, Iris Muller, Martin J. D. Clift

**Affiliations:** 1 Swansea University Medical School, Swansea University, Singleton Park, Swansea SA2 8PP, UKs.h.doak@swansea.ac.uk (S.H.D.); k.m.hawkins@swansea.ac.uk (K.H.); 2 Unilever, Safety and Environmental Assurance Centre, Colworth Science Park, Sharnbrook, Bedfordshire MK44 1LQ, UK; hugh.barlow@unilever.com (H.J.B.); iris.muller@unilever.com (I.M.)

**Keywords:** cell migration, vasculature, sprouting, endothelial cells, biomaterial, fibrin

## Abstract

A hallmark of angiogenesis is the sprouting of endothelial cells. To replicate this event in vitro, biomaterial approaches can play an essential role in promoting cell migration. To study the capacity of a scaffold of fibrin (fibrinogen:thrombin mix) to support the movement of the endothelial cells, the migration area of spheroids formed with the HULEC cell line was measured. The cells were first allowed to form a spheroid using the hanging drop technique before being encapsulated in the fibrin gel. The cells’ migration area was then measured after two days of embedding in the fibrin gel. Various conditions affecting fibrin gel polymerization, such as different concentrations of fibrinogen and thrombin, were evaluated alongside rheology, porosity, and fiber thickness analysis to understand how these factors influenced cell behavior within the composite biomaterial. Data point toward thrombin’s role in governing fibrin gel polymerization; higher concentrations result in less rigid gels (loss tangent between 0.07 and 0.034) and increased cell migration (maximum concentration tested: 5 U/mL). The herein presented method allows for a more precise determination of the crosslinking conditions of fibrin gel that can be used to stimulate angiogenic sprouting.

## 1. Introduction

Blood vessel formation in vivo is achieved by two processes: vasculogenesis and angiogenesis. In adults, vasculogenesis occurs when endothelial progenitor cells migrate from the bone marrow to the target site and form de novo blood vessels. In contrast, during angiogenesis, blood vessels are formed from pre-existing ones, where in the beginning, endothelial cells differentiate into tip and stalk cells. The tip cells develop filopodia and define the direction of the new blood vessel, while the stalk cells, with a high proliferative profile, follow them, forming the cord. The cord later develops a lumen that is perfused before final vessel maturation by pericytes or smooth muscle cells. All these actions are coordinated with basement membrane degradation [1]. To create viable vasculature models in vitro, it is crucial to replicate these coordinated actions and cellular behaviors. However, the lack of integrated vasculature models is a major limitation in the field of in vitro modeling. To accurately mimic physiological oxygen conditions, cells should be located within 100 µm of a capillary, ensuring access to a high oxygen source [2]. For instance, near the alveoli’s capillaries, this corresponds to 100 mmHg of oxygen partial pressure; however, at the tissue level, it can drop to 40 mmHg. In the in vitro setting, the oxygen concentration of a humidified incubator (5% CO_2_ and 37 °C) is 18.6%, which at sea level corresponds to an oxygen partial pressure of 141.1 mmHg (or 7.23 mM). However, this is still far away from the oxygen concentration that the cells would be exposed to since oxygen consumption rate and media height determine the oxygen gradient [3]. As we transition to three-dimensional tissue models, maintaining an adequate oxygen supply becomes even more challenging, as oxygen diffusion into the scaffold must be considered. The inability to establish a functional vasculature network within lab-based tissue analogs restricts their thickness, as nutrients and oxygen can only reach cells through diffusion. To overcome this limitation, various strategies have been explored, including the use of inserts, organoids, biomaterials, organ-on-a-chip technologies, and bioprinting (Table 1).

In the present study, a fibrin gel was selected as the biomaterial to support angiogenesis. Fibrin is the product of a reaction between fibrinogen and thrombin, which forms the basis of blood clots. After the formation of a blood clot in vivo, the endothelial cells degrade it in the process of wound healing. During this phase, biochemical factors released during the biological degradation stimulate angiogenesis in a positive feedback loop [4,5]. This makes the use of a fibrin gel a physiologically relevant biomaterial and enables angiogenesis per se. However, angiogenesis is not only regulated by soluble factors [6,7] but also by the mechanical properties of the extracellular matrix. The fibrin matrix rigidity has an impact on the progression of the different stages of the angiogenic process [8]. In this way, to create a better in vitro vasculature network, both factors need to be taken into consideration. This paper proposes a method to screen for the fibrin polymerization condition that could help narrow down the optimal crosslink conditions to support endothelial cell migration, a key feature of sprouting.

**Table 1:** Review of currently available strategies to form vasculature in vitro. Legend: ES: external stimulation; M: media perfusion; -: absence of external stimulation; hMSC: human mesenchymal stem cells; HUVECs: human umbilical vein endothelial cells; EC: endothelial cells; fibrin: blood clot mimicking when adding fibrinogen and thrombin; PCL: Polycaprolactone; PLGA: poly(lactic-co-glycolic acid).

Model Type	Overview	Cells	Matrix/Design	ES	Ref.
Inserts	Test of topographical and biochemical cues in endothelial barrier	HUVECs	Collagen-coated PET-transwell membrane electrospinning with PLC nanofibers	-	[9]
Organoid	Organoid formation for tumor cell Migration studies	Primary EC and A549 lung cancer cells in 3:1 ratio in methylcellulose	Dispersed fibroblasts in fibrin	-	[10]
(Bio)scaffolds	Test diffusion limits in a thick vascularized model	Fibrin gel populated with dispersed fibroblasts and beads coated with HUVECs		-	[11]
	Test MSC role in angiogenesis	Fibrin gel populated with dispersed MSC and beads coated with HUVECs		-	[12]
	Normal and tumor vasculature formation	HUVECs, smooth muscle cells and fibroblasts, MSC, HepG2 cell line	starPEG-heparin hydrogels	-	[13]
Organ-on-a-chip	PDMS device for vasculature formation studies	Primary EC and fibroblasts	PDMS device coated with fibrin	M	[14]
	Study of angiogenesis and thrombosis in a microvessel	HUVECs pericytes	PDMS reservoir filled with collagen with dispersed pericytes.	M	[15]
3D bioprinting	3D lattice structure resembling vasculature network	hMSC HUVECs	Vasculature ink: Pluronic F-127 and thrombinCell ink: gelatin, fibrinogen, and cells	M	[16]

Based on the above rationale, we investigated how the concentration of thrombin and fibrinogen affected clot formation regarding gel rigidity, gel porosity, and fiber diameter. Fibrinogen physiological value in the blood is between 2.0 and 4.5 mg/mL, considering a critical concentration below <1.0 mg/mL [17]. For thrombin, the value in the blood is between 5 and 10 nM [18]. Therefore, the fibrinogen concentrations selected for this study were 1.25, 2.5, and 5 mg/mL, and for thrombin, 1, 10, and 50 nM, which were converted using the supplier’s characteristic units/mg to 0.1, 1, and 5 U/mL, respectively. Additionally, the capacity of a healthy human endothelial cell line (HULEC cell line) to migrate inside the gel was also determined. To evaluate the capacity of the endothelial cells to migrate across the fibrin gel, the cells were seeded to form a spheroid. Afterward, fibrinogen and thrombin were pipetted, and the spheroid was embedded in the fibrin gel as it underwent polymerization. The capacity of the cells to release from the spheroid and migrate inside the fibrin gel was subsequently monitored using a bright field microscope. A similar experimental design was published before to test the capacity of cancer cells to invade tissues, which is considered a key hallmark of malignant tumors [19,20,21]. In this work, a similar approach was applied to test the capacity of the HULEC line cells to migrate inside the fibrin gel, a hallmark for angiogenesis. In this way, in this work, the gel’s rheological properties and fiber network morphology were correlated with cell migration within the fibrin gel. This aimed to identify the mechanical properties that best facilitate cell migration.

## 2. Materials and Methods

### 2.1. Part 1: 2D Cell Characterization

#### 2.1.1. Cell Culture

The immortalized human lung microvascular endothelial cell line HuLEC-5a (ECs) was isolated from healthy human lung microvasculature (ATCC^®^, #CatCRL3244). The ECs were cultured in MCDB131 (Gibco, #Cat10372019) media supplemented with 10% Fetal Bovine Serum (FBS, Gibco, #Cat16140071), 10 ng/mL of human epidermal growth factor (Merk, #CatE9644), 1 µg/mL of hydrocortisone (Merk, #CatH4001), 10 mM of L- Glutamine (Gibco, #Cat25030-024), and 1% Penicillin/Streptomycin (Gibco, #Cat15140-122). Hydrocortisone was always added fresh to the media. The ECs were used for experimentation from passage 3 to passage 8.

#### 2.1.2. Endothelial Cells 2D Cell Characterization

For all assays presented, the ECs were seeded at 5000 cells/cm^2^ in lab plasticware (flasks and plates), and the characterization lasted 7 days.

- Cellular viability

Cell viability was determined using the Trypan Blue Exclusion assay (Gibco, Cat#11538886). The cells were detached with trypsin (Gibco, Cat#25300062), and 10 µL of the cell suspension was added to 10 µL of trypan blue solution (0.4%). Then, the live and dead cells were counted using a hemocytometer via the trypan blue exclusion method as previously described [22];

- Metabolic activity

This parameter was determined using the MTS assay, as per the manufacturer’s guidelines (Promega, #CatG3582). Initially, the media was removed from the wells, and fresh media (without FBS) with MTS at a ratio of 5:1 were added. Then, the cells were placed in the incubator (humidified at 37 °C with 18% oxygen and 5% CO_2_) for 1 h for the reaction to occur. After the incubation period, the media were removed and added to a new 96-well plate. The absorbance was measured by a plate reader (POLARstar Omega, BMG LABTECH) at 490 nm. The negative control corresponded to a well where no cells were seeded. Subsequently, test sample absorbance values were subtracted from those of the negative control and divided by the plate area. The MTS value was further divided by the number of live cells per cm^2^, as previously determined by the trypan blue exclusion assay;

- Cell proliferation

The proliferation rate of ECs was determined using the Picogreen assay following the manufacturer’s guidelines (ThermoFisher Scientific, Cat#P7589). The cells were detached with trypsin and disrupted with Tris-EDTA buffer. Next, 100 µL of the cell lysate and standards were loaded into a black 96-well plate (Greiner, flat bottom). A total of 100 µL of the Picogreen dye solution diluted 200× in PBS was then added to each well. Immediately, the plate was analyzed using a fluorimeter (POLARstar Omega, BMG LABTECH), where it was first shaken for 5 min at room temperature prior to fluorescence measurement at 510–520 nm (excitation reference 485 nm). The ng of DNA in the samples was determined via a calibration curve (y = mx + b);

- CD31 surface marker

The surface marker CD31 was determined using flow cytometry with the ACEA Biosciences NovoCyte^®^ Flow Cytometer instrument (model number 3000). The cells were detached with trypsin, and a total of 1 × 10^5^ viable cells were diluted in 1 mL of PBS with 2.5 µL of CD31 (Biolegend, Cat#303103FITC) for 45 min protected from the light at 4 °C. Then, the cells were centrifuged for 5 min at 300 g; the supernatant was discharged, and 500 µL of flow cytometry buffer; PBS with 1% bovine serum albumin (BSA, Merk, Cat#A2153) and 0.1% sodium azide (Merk, Cat#S2002), was added. All analyses were compared to the negative control, which consisted of ‘cells only’, with no antibody staining added. Data analysis was performed using FlowJo 10.7.1 software (license number 59222). Firstly, 50,000 events were collected. The singlets were then gated by plotting the side scattering peak height (SSC-H) in function of the side scattering peak area (SSC-A) of the negative control. At this point, around 10,000 events were recorded. The singlet’s gate was then applied to all test samples to determine the number of events with and without fluorescence in a plot showing the SSC-A in the function of the FITC channel. The FITC fluorophore was excited at 488 nm, and a 530/30 filter was used to detect the signal;

- Cell morphology

Confocal microscopy (Carl Zeiss LSM 710) was used to assess all potential changes in cell morphology based on the cell culture environment. Cells were initially fixed with a 4% paraformaldehyde solution for 15 min at room temperature, followed by blocking the cells with a 5% FBS solution for 1 h at room temperature. Then, the primary antibody CD31 (Invitrogen, Waltham, MA, USA, #CatMA5-13188) was diluted in 1:100 in 1% BSA and incubated overnight at 4 °C in the dark. On the next day, the secondary antibody Goat anti-Mouse Alexa Flour Plus 488 (Invitrogen, #CatA32723) at 1:1000 dilution in 1% BSA was added together with the Phalloidin (Invitrogen, #CatA22284) at 1:200 dilution in 1% BSA for 2 h at room temperature in the dark. Afterward, DAPI (Merck, Rahway, NJ, USA, #CatD9542) was added at 1 µg/mL in PBS for 15 min at room temperature in the dark. For VE-cadherin staining, the same protocol was followed, but the VE-cadherin primary antibody (Cell Signaling Technology, Danvers, MA, USA, #Cat2500T) was added in a dilution of 1:400 in 1% BSA, and the secondary was the Alexa Flour 555 donkey anti-rabbit (Invitrogen, #CatA31572) at 1:1000. The 488 nm laser was used to excite the CD31, the 543 nm for the VE-Cadherin, the 405 nm for the DAPI, and the 633 nm for the Phalloidin.

### 2.2. Part 2: Acellular Fibrin Gel Characterization

#### 2.2.1. Rheologic Characterization

The rheological properties of the gels were measured using an AR-G2 Controlled Stress Rheometer (TA Instruments, New Castle, DE, USA). The geometry used during the measurements was a 40 mm parallel plate, Peltier plate steel. The gap between the plate and the rheometer bed was 78 µL. First, 39 µL of fibrinogen (Enzyme Research Laboratory, FIB 1) was pipetted on top of the rheometer bed, and then, 39 µL of thrombin (Enzyme Research Laboratory, HT1002a) was added on top of the fibrinogen drop. The plate was immediately lowered (to the gap of 78 µL) and rotated to ensure the fibrinogen and thrombin mixture. Then, data points were immediately collected using a continuous oscillation waveform (in direct strain mode) with 2% strain (deemed to be in the linear range of the sample) at 1.6 Hz and 37 °C. The measurements were allowed to continue until the storage (G′) and loss modulus (G″) reached the plateau, where it was left to record at least 1000 points used to perform the data analysis. The time taken to reach a plateau was recorded, and 1000 data points were analyzed to calculate the average final storage and loss modulus of the fibrin gel. For data analysis, the generated Excel sheet was imported into MATLAB R2019B (license number 92345). After plotting the graph of the storage and loss modulus over time, the time points of the plateau were identified, and 1000 selected points were averaged to calculate the storage and loss modulus of the fibrin gel (the MATLAB script is presented in Supplementary Material Figure S1). Each repetition corresponds to a single rheometer analysis, and an average of 3 to 6 independent rheometer characterizations were conducted for each polymerization condition. A total of nine distinct polymerization conditions were tested, varying fibrinogen concentrations at 5, 2.5, and 1.25 mg/mL and thrombin concentrations at 5, 1, and 0.1 U/mL.

#### 2.2.2. Confocal Microscopy

Equal volumes of fibrinogen and thrombin were added, making a total volume of 200 µL. A total of nine distinct polymerization conditions were tested, varying fibrinogen concentrations at 5, 2.5, and 1.25 mg/mL and thrombin concentrations at 5, 1, and 0.1 U/mL. To ensure fiber visualization, 4% of the fibrinogen was substituted by the 488-conjugated fibrinogen (ThermoFisher, #CatF13191). After gel formation in the hood, the gels were incubated for 24 h to ensure complete polymerization. Afterward, the gels were processed for paraffin embedding and sliced at 8 µm thickness. The slides were imaged on a Zeiss LSM 710 confocal microscope with a 488 nm laser excitation. For analysis of the gel’s porosity, the images were processed using ImageJ^TM^ (open source software; version 1.53K) batch process function (ImageJ script and representative image of the analysis can be found in Supplementary Material Figure S2). In brief, first, the script converted the color images into black and white to quantify black and white pixels that matched pores versus the fibers, respectively. A total of 2 to 3 gels were imaged per condition; from each gel, 3 microscope slides from three different parts of the scaffold were imaged (each slide was imaged at least 3 times). Each microscope slide was considered an independent repetition when calculating averages and performing statistics.

#### 2.2.3. Scanning Electron Microscopy

The slides were dewaxed for paraffin removal by washing them with xylene, followed by a gradient of 95%, 70%, and 50% ethanol. Each washing step took three minutes. Prior to drying the slides in the desiccator, they were rinsed in tap water to remove ethanol traces. The slides were coated with 2 nm chromium with a Q150TE coater from Quorum Technologies before being imaged on the Hitachi S-4800 Scanning Electron Microscope. A total of nine distinct polymerization conditions were tested, varying fibrinogen concentrations at 5, 2.5, and 1.25 mg/mL and thrombin concentrations at 5, 1, and 0.1 U/mL. For analysis of the fiber diameter, ImageJ^TM^ (open source software; version 1.53K) was used. In brief, the image was loaded into the program; then, the scale was added, followed by manual measurement of the fiber diameter with the free-hand tool. A total of 2 to 3 gels were imaged per condition; from each gel, 3 microscope slides from three different parts of the scaffold were imaged. In addition, each slide was imaged at least 3 times, and from each image, 3 to 5 fibers were measured. Each microscope slide was considered an independent repetition when calculating averages and performing statistics.

### 2.3. Part 3: Endothelial Cell Migration

To evaluate the capacity of the endothelial cells to migrate across the fibrin gel, the cells were seeded to form a spheroid. Afterward, fibrinogen and thrombin were pipetted, and the spheroid was embedded in the fibrin gel as it polymerized. Then, the capacity of the ECs to come out of the spheroid and migrate in the fibrin gel was monitored using a bright field microscope.

#### 2.3.1. Spheroids Formation

Spheroids were initially formed by the hanging drop technique, which consisted of pipetting a 20 µL drop of complete MCDB131 media, with 10% FBS extra supplementation and 3000 cells on the lid of a 48-well plate. After 3 days in the incubator (humid 37 °C, 18% oxygen and 5% CO_2_), the spheroids were formed. Before proceeding to spheroids embedding in fibrin gel, bright field pictures were captured using the Olympus BX51 to evaluate spheroid geometry. Only round and intact spheroids were considered for further embedding. In total, *n* = 5, each repeat consisting of an average of 2–4 spheroids.

#### 2.3.2. Spheroids Fibrin Encapsulation

To start, the plate was flipped, and to the 20 µL drop where the spheroid was formed, 10 µL of fibrinogen and 5 µL of thrombin were added. The plate was left undisturbed for 15 min in the tissue culture hood before being moved to the incubator (humidified at 37 °C, 18% oxygen, and 5% CO_2_). After 24 h, a silicone ring with a diameter of 8 mm was placed around each spheroid to contain 100 µL of complete MCDB131 media. On the second day after embedding, the media were removed from the rings and added to a 4% paraformaldehyde solution to fix the spheroids for 30 min. Then, the spheroids were stained with hematoxylin, Hoechst, and Propidium Iodide. In total, five independent experiments were performed, with each repeat consisting of an average of 2–4 spheroids.

##### Hoechst and Propidium Iodine

After spheroid fixation, 100 µL of a staining solution containing 30 mg/mL of Hoechst 33342 Trihydrate (Life Technologies, #CatH3570) and 30 mg/mL of Propidium Iodide (Merk, #CatP4864) was added to each spheroid. The solution was incubated for 30 min at room temperature in the dark. Afterward, the spheroids were washed three times with PBS and imaged on a ZEIS LSM 710. The lattice function in the microscope was used to image the entire spheroid. In total, five independent experiments were performed, with each repeat consisting of an average of 2–4 spheroids.

After Hoechst and Propidium Iodide (PI) imaging, 100 µL of hematoxylin (Merk, #CatHX99311375) solution was added to each spheroid for 10 min at room temperature. Afterward, the spheroids were washed with PBS until no traces of dye were present in the washings. Then, the spheroids were imaged on an Olympus BX51 microscope (software cellsens). For data analysis, the images were loaded on ImageJ software. Then, using the free-hand tool, the initial area of the spheroid was outlined, followed by the total area of cell migration. Afterward, the migration area was determined by subtracting the spheroid area from the area of cell migration (Supplementary Material Figure S3 shows an example of a spheroid analysis in ImageJ). In total, five independent experiments were performed, with each repeat consisting of an average of 2–4 spheroids.

### 2.4. Data Presentation and Statistical Analysis

Raw data were handled using Microsoft Office Excel 365. The arranged data were then exported to GraphPad Prism version 8.4.3 to generate graphs and perform the statistical analyses (serial number: GP8-1621658-R). The data are presented as the mean ± standard deviation (SD). The data were analyzed with ANOVA. Ordinary two-way ANOVA used Tukey’s multiple component test or Sidak’s multiple component test. One-way ANOVA used the Friedman Test and Dunn’s multiple comparison test. The significance values were taken when *p* < 0.05, graphically denoted as * *p* < 0.05, ** *p* < 0.01, *** *p* < 0.001. **** *p* < 0.0001. If the data in the graph do not contain an asterisk, it is because they are not significant.

## 3. Results

### 3.1. Part 1: 2D Cell Characterization

**Figure 1:**
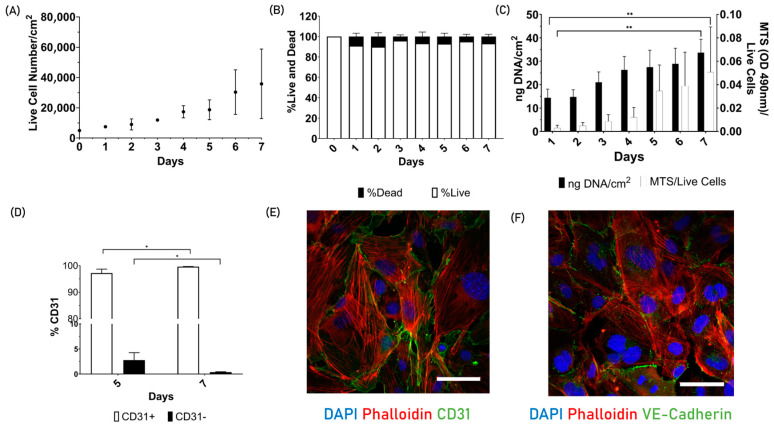
Endothelial cell 2D characterization. (**A**) Live cell quantification with trypan blue over 7 days. The cell growth fits the following exponential curve: y = 5381e0.2741x. (**B**) Live and dead cell ratio using trypan blue assay. (**C**) Picogreen dsDNA quantification over 7 days on the left y-axis. The increase in dsDNA is statistically significant from day 1 to 7 with a *p*-value of 0.0070. The right y-axis corresponds to the MTS absorbance over the number of live cells. The cell’s metabolism increases from day 1 to 7 and is also statistically significant, with a *p*-value of 0.0040. (**D**) Expression of the CD31 marker on days 5 and 7. The increase in CD31 surface marker from day 5 to 7 is statistically significant, with a *p*-value of 0.044. This is associated also with a decrease in CD31, with a *p*-value of 0.044. (**E**) Endothelial cells on day 7 of the cell culture stained with DAPI, Phalloidin, and CD31 (scale bar represents 50 µm). (**F**) VE-Cadherin (scale bar represents 50 µm). The significance values were taken when *p*-No value < 0.05, graphically denoted as * *p* < 0.05 and ** *p* < 0.01. The error bars correspond to the Standard Deviation. *n* = 3.

To understand the nature of the endothelial cells, they were first characterized in 2D (Figure 1). Cells showed exponential growth over 7 days (Figure 1A). The curve fitted is the following: y = 5381e0.2741x, with an initial value of 5381 and a constant rate of 0.2741 day-1. With these data, a doubling time of 2.5 days was determined for these cells in 2D culture. The trypan blue exclusion assay data were used to generate a similar graph (Figure 1B), which shows the live and dead cell ratio. During the seven-day experimental period, the percentage of dead cells remained below 10%. The highest values were recorded on days 1 and 2, with 8% and 9%, respectively. Another indication that the cells were undergoing division and normal homeostasis during the seven days was the statistically significant increase in dsDNA (Figure 1C). Alongside the dsDNA data, the MTS values are presented, showing that the metabolic activity per number of live cells also increased over the seven-day testing period. Furthermore, Figure 1D shows the percentage of CD31 quantified via flow cytometry, where from day 5 to day 7, the number of cells expressing CD31 increases, with a corresponding decrease in CD31-negative cells. In addition, Figure 1E,F provides a detailed characterization of cell morphology on day 7. The cells showed confluency and no changes in cell morphology over time. Additionally, the green fluorescence indicates the specific surface markers CD31 (cell–cell adhesion protein) and VE-cadherin (vasculature endothelial cadherin) localized preferentially on the cell membrane, which is characteristic of endothelial cell phenotypes.

### 3.2. Part 2: Acellular Fibrin Gel Characterization

To better understand the physical environment that endothelial cells prefer for migration, the fibrin gel network was characterized in terms of its rheological properties, network morphology, porosity, and fiber thickness.

#### 3.2.1. Rheologic Characterization

**Figure 2:**
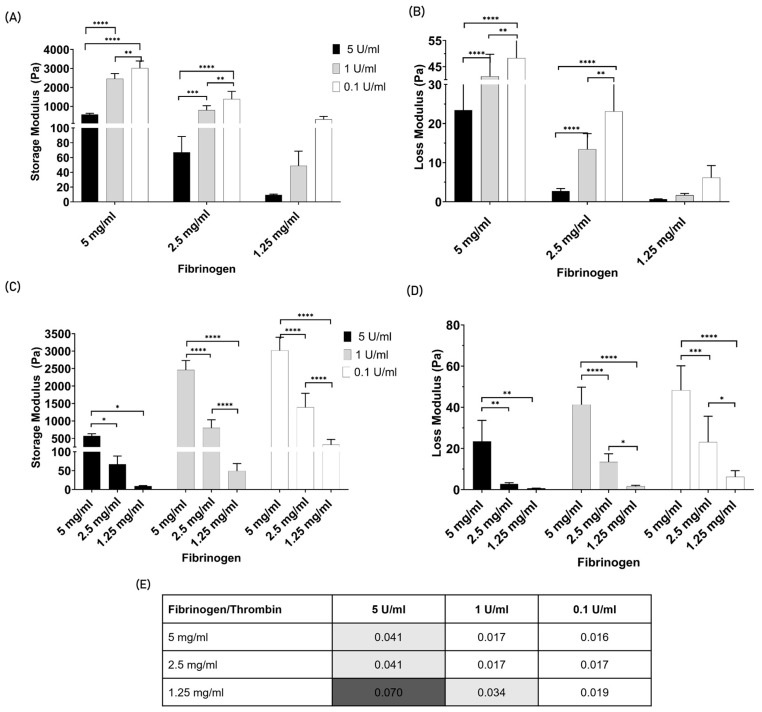
Fibrin gel rheological characterization. (**A**) Storage (G′) and (**B**) Loss modulus (G″) of fibrin gel in Pascal (Pa). The data are grouped by the fibrinogen concentration. (**C**) Storage (G′) and (**D**) Loss modulus (G″) of fibrin gel in Pascal (Pa). The data are grouped by the thrombin concentration. (**E**) The table below shows the loss tangent values. The color scheme highlights values in the same range. The fibrinogen concentrations tested were 5, 2.5, and 1.25 mg/mL, and for thrombin, they were 5, 1, and 0.1 U/mL. The error bars correspond to the Standard Deviation. The data are between *n* = 3 and 6. The significance values were taken when *p* < 0.05, graphically denoted as * *p* < 0.05, ** *p* < 0.01, *** *p* < 0.001 and **** *p* < 0.0001.

To gain a deeper understanding of the mechanical environment that favors endothelial cell migration, the rheology of the final fibrin gel network was analyzed. Figure 2 shows the rheology (i.e., storage and loss modulus) of the fibrin gels from the nine polymerization conditions tested. Figure 2A,B shows the storage and loss modulus values grouped by the fibrinogen condition, and in Figure 2C,D, the same data are grouped by the thrombin concentration to facilitate data readability. In addition, Figure 2E shows the loss tangent values. For all the fibrin gel polymerization conditions, the storage modulus final values are much higher than those of the loss modulus, indicating that gelation efficiently occurred. Changing the thrombin concentration from 0.1 to 1 and 5 U/mL within the same fibrinogen concentration resulted in a decrease in both the loss and storage moduli. In contrast, as the fibrinogen concentration increased from 1.25, 2.5, to 5 mg/mL, both the storage and loss moduli increased. However, to better understand the relationship between the two moduli, the loss tangent was determined (Figure 2E). This variable corresponds to the ratio between the loss and storage moduli during each cyclic deformation. In all tested conditions, the ratio is much less than one, meaning that the elasticity dominates the viscous properties of the fibrin gel, with a magnitude ranging from 14 to 62 times higher. This finding is indicative of ‘solid-like’ behavior due to gel polymerization. From the analysis of Figure 2E, it can be concluded that despite the increase in fibrinogen leading to an increase in the storage and loss moduli, the loss tangent decreases, indicating that the storage modulus increases relatively more than the loss modulus. On the other hand, thrombin increase is associated with a decrease in both storage and loss moduli; however, the loss tangent increased, concluding that the storage modulus increased faster than the loss modulus. This indicates that both phenomena, an increase in fibrinogen or a decrease in thrombin, contribute to more rigid gels. Notably, for the fibrinogen concentration of 1.25 mg/mL, the increase in loss tangent is linear with the increase in thrombin. However, this linear increase is not observed for the fibrinogen concentrations of 2.5 and 5 mg/mL. In addition, the values of Figure 2E are highlighted in greyscale to better visualize the fibrinogen/thrombin polymerization conditions that have the same value of loss tangent, hence indicating comparable rigidity levels. The dark grey highlights the softest gel condition (5 mg/mL fibrinogen crosslink with 5 U/mL thrombin), while the conditions highlighted in a light grey share intermediate rigidity. The remaining conditions represent the most rigid gels.

#### 3.2.2. Confocal Microscopy

**Figure 3:**
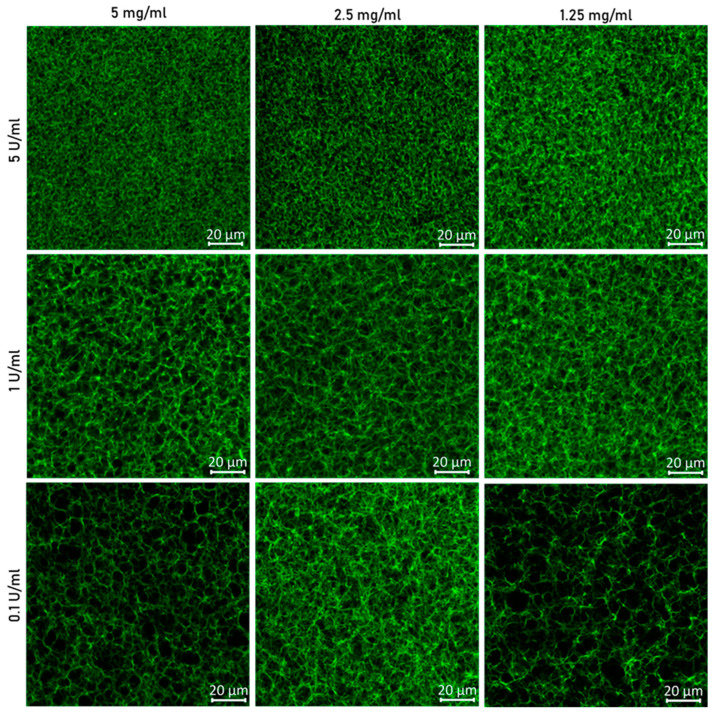
Confocal microscope images of fibrin gel polymerized with 5, 2.5, and 1.25 mg/mL of fibrinogen and 5, 1, and 0.1 U/mL of thrombin. The fibrinogen solution was mixed with 4% of fibrinogen from human plasma Alexa Fluor 488 conjugated to allow for fiber visualization when excited with a 488 laser. The scale represents 20 µm. *n* = 6 to 9.

To further characterize the fibrin gel network, fluorescently labeled fibrinogen was mixed with regular fibrinogen, highlighting in green the fiber density on a macroscopic scale (Figure 3). The most noticeable effect is observed with thrombin; reducing the thrombin concentration resulted in larger pore sizes and longer fibers.

#### 3.2.3. Scanning Electron Microscopy

**Figure 4:**
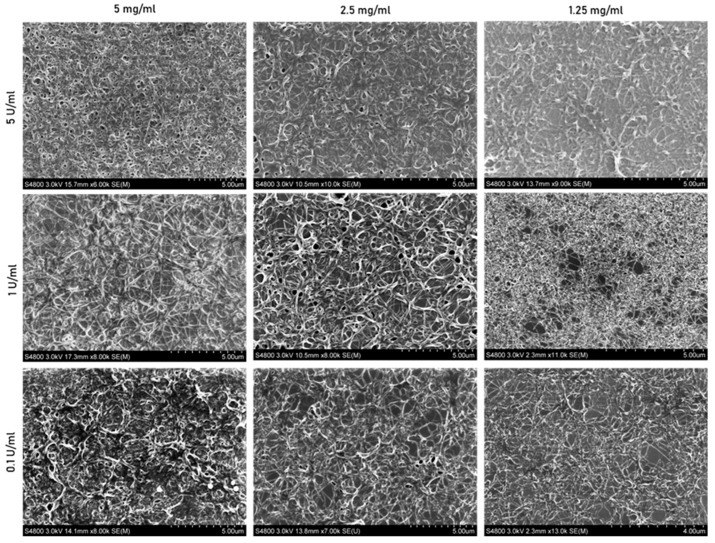
Scanning electron microscope images of fibrin gel polymerized with 5, 2.5, and 1.25 mg/mL of fibrinogen and 5, 1, and 0.1 U/mL of thrombin. The scale bar represents 5 µm, except for condition 1.25 mg/mL with 0.1 U/mL, which represents 4 µm. *n* = 3.

The scanning electron microscope (SEM) images in Figure 4 provide a detailed view of the fibrin fiber surface topography, capturing their arrangement and entanglement under various polymerization conditions. These images offer a closer look at the fiber architecture and dimensions with a 5 µm scale bar compared to the broader perspective in Figure 3, which uses a 20 µm scale bar to show fiber density. The SEM images provide additional insights into the polymerization process by enabling the measurement of the fiber’s diameter.

#### 3.2.4. Porosity and Fiber Thickness

**Figure 5:**
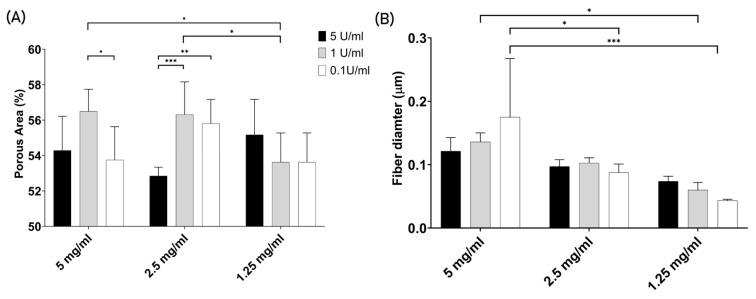
Fibrin gel porosity and fiber diameter. (**A**) Percentage of the area corresponding to pores in the fibrin gel in function of fibrinogen and thrombin concentration (*n* = 6 to 9). (**B**) Fiber diameter in micrometers (µm) in function of fibrinogen and thrombin concentration (*n* = 3). The significance values were taken when *p* < 0.05, graphically denoted as * *p* < 0.05, ** *p* < 0.01, and *** *p* < 0.001. The error bars represent the Standard Deviation.

It was proposed that changes in polymerization conditions would lead to variations in the gel’s porosity, thereby impacting the migratory capacity of the cells. In this way, the confocal microscopy images presented in Figure 3 were further processed to determine the gel’s porosity (Figure 5A). However, Figure 5A demonstrates that the porosity of the gels does not vary significantly among the nine tested polymerization conditions, with values ranging from 56.5% to 52.8%. In addition, the data do not show any trend across the same conditions of thrombin or fibrinogen. The fiber thickness was also determined, with SEM images from Figure 4 being further analyzed to measure the fiber diameter (Figure 5B). The plot shows a decreased tendency in fiber thickness as fibrinogen concentration decreased, meaning that the fiber diameter dropped as the fibrinogen concentration also decreased from 5, 2.5 to 1.25 mg/mL. Concerning the impact of thrombin on fiber diameter, the trend is not consistent across all fibrinogen concentrations. At the highest concentration of 5 mg/mL, an increase in thrombin results in a decrease in fiber diameter. Meanwhile, for the 2.5 mg/mL concentration, the values remain similar to each other, with a diameter of around 0.1 µm. At the lowest fibrinogen concentration tested (1.25 mg/mL), the fiber diameter increased with higher thrombin levels. 

### 3.3. Part 3: Endothelial Cell Migration

**Figure 6:**
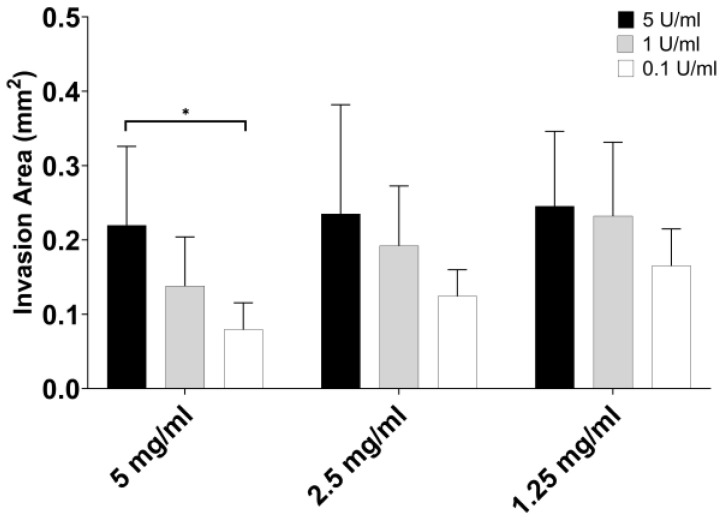
Endothelial cell migration area in function of the fibrinogen and thrombin polymerization conditions. The asterisk symbol in the plot represents * *p* < 0.05. The error bars represent the Standard Deviation. *n* = 5.

To assess the suitability of each fibrin gel polymerization condition in supporting cell migration, the endothelial cells were cultured into spheroids and subsequently embedded in the nine different polymerization conditions. After two days, the migration area was evaluated (Figure 6). A visual trend is observed in the data, indicating that a higher thrombin concentration leads to an increased migration of endothelial cells, regardless of the fibrinogen concentration. The higher capacity to travel inside the gel was found when the thrombin concentration was 5 U/mL, independent of the fibrinogen concentration. Another condition that stands out is 1.25 mg/mL of fibrinogen crosslink with 1 U/mL of thrombin, with a migration area in the range of 0.2 mm^2^. Looking at the fibrinogen influence and fixing the thrombin concentration at 5 U/mL, the migration area was similar for all conditions of fibrinogen. Then, fixing for 1 U/mL or 0.1 U/mL, the migration area increases when fibrinogen concentration decreases from 5 to 1.25 mg/mL.

**Figure 7:**
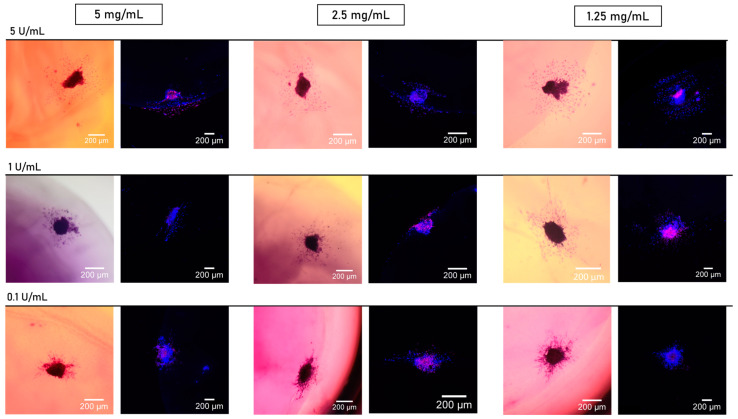
Spheroid bright and fluorescence staining. Spheroids on day 2 after fibrin embedding. The column on the left represents hematoxylin stain, and on the right, Hoechst and PI. The scale bar represents 200 µm. *n* = 5.

To better illustrate the results in Figure 6, a representative image of the hematoxylin-stained spheroids is provided in Figure 7. These images were used to generate the plot in Figure 6. Spheroids stained with hematoxylin reveal cells migrating from the initial spheroid structures, with a higher migration area observed in spheroids polymerized with 5 U/mL of thrombin. A decrease in thrombin concentration corresponded to a reduction in the migration area. Additionally, Figure 7 shows the spheroids stained with Hoechst to visualize live cells and PI to visualize dead cells. The non-viable cells are more concentrated toward the center of the spheroid.

## 4. Discussion

The ability of a biomaterial to support cell migration is crucial for promoting sprouting during angiogenesis. This study introduces a method to screen various polymerization conditions of fibrin gel and identifies the ones that most effectively promote cell movement. This method was proven before using Matrigel to show cancer invasion, a key hallmark of malignancy [19,20,21].

The 2D characterization of the HULEC cell line shows that the cells are viable and express specific surface markers of endothelial cells, indicating the suitability of this cell line for assessing cell migration in the fibrin gel. In addition to the 2D data, the cells were also seeded in the fibrin gel to ensure cell viability and proliferation when embedded in the gel. Data in Supplementary Material Figure S4 show cell proliferation over 21 days for the different fibrinogen and thrombin concentrations tested in this work. These preliminary experiments provide insights into the suitability of the gel environment for supporting cell viability and function before testing its migration capacity.

The clot formation (fibrinogen and thrombin crosslink) in static conditions will occur until all soluble fibrinogen is converted into insoluble fibrin. Two phenomena influence polymerization: lateral aggregation and branching of the fibers. The fibrin fibers thicken and elongate by lateral aggregation. However, if fibrinogen is cleaved at a fast rate, lateral and length fiber growth is slower compared to branching. This results in a network of thin and short fibers and gels with higher density and smaller pores, while, if later aggregation dominates the reaction, fibrin fibers thicken, branching points are reduced, and it creates gels with larger pores. The competition between branching and lateral aggregation depends on the thrombin activity; higher thrombin activity promotes branching; on the contrary, low thrombin activity promotes lateral aggregation [23,24]. The theory explained before is aligned with the images from Figure 3. The conditions with higher thrombin concentration show gels with higher branching density, reduced fiber length, smaller pores, and fiber thinning. Higher fibrinogen concentrations are also responsible for increasing the number of monomer formations, contributing to the acceleration of the branching reaction [23]. Despite that, the thrombin concentration dominates the polymerization reaction and defines the fibrin network morphology.

Despite visually observing the impact of thrombin on the fibrin network, the porosity data did not provide definitive conclusions. It is important to highlight that, in this study, we did not assess the dimensions of the pores; instead, our focus was on determining the percentage of pixels associated with either pores or fibers. This area only slightly varies in percentage between the nine polymerization conditions and does not show any trends across the same values of fibrinogen or thrombin. This conclusion was also observed when subjecting the data to the other segmentation algorithms from ImageJ, such as the Default and the Percentile (data on Supplementary Material Figure S5). The three algorithms vary on the threshold criteria when identifying the pixel associated with fiber and the pixel associated with pores. This has an impact on the percentage of areas allocated to pores or fiber. However, from the three algorithms, it is not possible to have a trend between the different conditions of thrombin and fibrinogen, which may indicate that a balance between a network of thicker fibers with bigger pores and thin fibers, and smaller pores may lead to the same area occupied by fibers.

The mechanical properties of fibrin indicate that the gels behave as a viscoelastic polymer. This means that the gel assumes a behavior between an ideal elastic material (Hooke’s Law) and a viscous material (Newton’s Law of Viscosity). The elasticity, or rigidity, is characterized by a reversible mechanical deformation; when the stress is removed, the material returns to its original position. The viscosity is characterized by an irreversible mechanical deformation; all energy applied is dissipated in the flow of the material. The elastic component of the gel is measured by the storage modulus. The loss modulus measures the viscous component of the material. A higher storage modulus value means that a larger force is necessary to be applied to the gel surface to produce a displacement; the gel is stiffer or rigid. Essentially, it has a greater ability to maintain its shape and support. A clot exhibiting a higher loss modulus will lose more energy deforming, indicating that it is less able to maintain its shape and may exhibit more fluid-like behavior. It is associated with softness [25]. In simpler terms, a fibrin gel with a high storage modulus is firmer and more solid-like, while a fibrin gel with a high loss modulus is more fluid-like, less firm, and softer. However, to easily analyze the magnitude of the elastic and viscous components in a gel, the ratio between the loss and storage modulus (given by the loss tangent) is determined. This ratio (i.e., Figure 2E) shows that despite the increase in fibrinogen leading to an increase in both moduli, the storage modulus increases faster than the storage modulus, leading to a decrease in the loss tangent. The decrease in this variable is associated with more rigid gels, concluding that within the polymerization conditions used, the higher fibrinogen concentration is associated with more rigid gels. In addition, fibrin gels with more fibrinogen present higher fiber diameter, more branching points, lower fiber length, and smaller pores. Regarding its suitability to support cellular mobility, the results depend on the thrombin concentration. For 5 U/mL of thrombin, the concentration of fibrinogen did not significantly change the migration area. However, for 1 and 0.1 U/mL of thrombin, the cells preferred to migrate in gels polymerized with less concentration of fibrinogen. Indicating that the thrombin is responsible for the results when concentration is high, but if thrombin concentration is low or has less strength (less U/mL per gram of product), the concentration of the fibrinogen has a higher impact, and the cells prefer less fibrinogen, which results in less rigid gels. 

Regarding the thrombin effect, an increase in this factor leads to softer gels, as it is associated with higher loss tangent values. However, the increase in loss tangent is higher when thrombin increases from 1 U/mL to 5 U/mL. The increase in thrombin also leads to a visual increase in branching points, lower fiber length, and smaller pores, similar to what was observed with the fibrinogen. The fiber diameter does not exhibit a clear tendency; depending on the fibrinogen concentration, the diameter shows a different trend. For this variable, it was not possible to find an explanation for the data obtained. However, for the cellular migration area, the trend is significant, where higher thrombin levels during crosslinking result in fibrin gels that support higher cell migration. In this way, a good correlation between cell migration and gel rigidity was found. The polymerization conditions showed higher cell migration when 5 U/mL of thrombin was mixed with 5, 2.5, or 1.25 mg/mL of fibrinogen, as well as when 1.25 mg/mL polymerized with 1 U/mL. All these conditions are highlighted in dark and light grey in Figure 2E, which is associated with higher loss tangent values (values between 0.07 and 0.034), indicating softer gels as well.

Regarding network morphology, Esther and colleagues [23] show that the increase in thrombin leads to an increase in fiber density and branching points, with fibers exhibiting shorter lengths and smaller diameters. However, the thinning of the fibers in the work presented here was only observed when 5 mg/mL of fibrinogen was used. 

The gel’s rheological behavior was also recorded in Esther et al. [23], showing similar results to the ones presented here for the fibrinogen. Gels polymerized with more fibrinogen show higher values of storage and loss modulus and a decrease in loss tangent. The authors also show that an increase in thrombin is associated with a decrease in loss and storage modulus as well as a decrease in the loss tangent. This is contrary to the results presented here, and no other work in the literature found similar results for thrombin. The origin of the fibrinogen and thrombin greatly influences the results. The thrombin and fibrinogen clotting abilities differ in each manufacturer, which could explain the different results. 

Another important variable during polymerization is the calcium concentration. When more calcium is present, the gels exhibit a network with less fiber density, fewer branching points, and fibers with higher diameter and length. Also, the highest gel rigidity is exhibited between 1.5 and 2 mM of calcium, which corresponds to physiological blood calcium levers [23]. In the work described in this paper, the effect of calcium concentration was not explored. The calcium level was maintained constant and is given by the calcium content in the media in the form of calcium chloride. The concentration of this salt in the media is 1.6 mM (235 mg/mL); however, during polymerization, the media is diluted 1:2, so half of the concentration is present during crosslink. 

## 5. Conclusions

The methodology presented here to quantify the migration area of the spheroid is a cost-effective and non-laborious method to obtain information on a biomaterial’s ability to promote cell migration. This is an important aspect, especially when screening for a material that promotes sprouting associated with angiogenesis. In addition, the herein presented approach can be used as a platform to test angiogenic factors as well as different cell types to determine which one (or more) is most suitable for replicating angiogenesis in vitro. These data associated with the specific hydrogel characterization can inform a more efficient and effective approach toward determining optimal crosslinking conditions when tuning biomaterials for vasculature-based research and applications.

## Data Availability

The original contributions presented in the study are included in the article/Supplementary Material, further inquiries can be directed to the corresponding author.

## Supplementary Materials

The following supporting information can be downloaded at https://www.mdpi.com/article/10.3390/jfb15090265/s1, Figure S1: (**A**) ImageJ script applied in batch to convert the fluorescent fibrin images into black and white pixels. (**B**) Representative image of the condition 2.5 mg/mL of fibrinogen and 0.1 U/mL of thrombin with 4% 488-labelled fibrinogen before and after script application on the left and right respectively. The scale bar represents 10 µm; Figure S2: (**A**) MATLAB script applied to the rheometer data. (**B**) Example of MATLAB script output for loss (lm) and storage modulus (sm) for the condition tested with 2.5 mg/mL fibrinogen and 5 U/mL thrombin; Figure S3: ImageJ analysis of endothelial cell migration area. The left image shows a representative spheroid stained with haematoxylin, with its initial area outlined in yellow. The right image shows the same spheroid, highlighting the total area of cell migration, also outlined in yellow. The condition depicted corresponds to fibrin polymerization at 1.25 mg/mL + 1 U/mL; Figure S4: Proliferation of endothelial cells seeded inside fibrin gel over 21 days and regarding the different fibrinogen and thrombin concentrations. The cells were seeded in 100 µL fibrin gel in 96 well plates at 25,000 cells/gel. The error bars represent the Standard Deviation. *n* = 4; Figure S5: Percentage of area corresponding to pores in the fibrin gel depending on fibrinogen and thrombin concentration. Plot on the left corresponds to the data analyzed with the Default algorithm from ImageJ and plot o the right with the Percentile. The error bars represent the Standard Deviation. *n* = 6 to 9.
