# Supplementary material for: Exploring the Role of Fibrin Gels in Enhancing Cell Migration for Vasculature Formation
Source: J Funct Biomater. 2024 Sep 12;15(9):265. doi: 10.3390/jfb15090265 (PMC11432887; doi:10.3390/jfb15090265)
Supplement: Supplementary file 1 [file jfb-15-00265-s001.zip › jfb-3161776-supplementary.pdf]

(A)

```
setBatchMode(true);
run("8-bit");
run("Bandpass Filter...", "filter_large=160 filter_small=3 suppress=None tolerance=5 autoscale saturate");
setAutoThreshold("Huang");
//run("Threshold...");
setAutoThreshold("Huang");
setOption("BlackBackground", false);
run("Convert to Mask");
run("Measure");
```

(B)

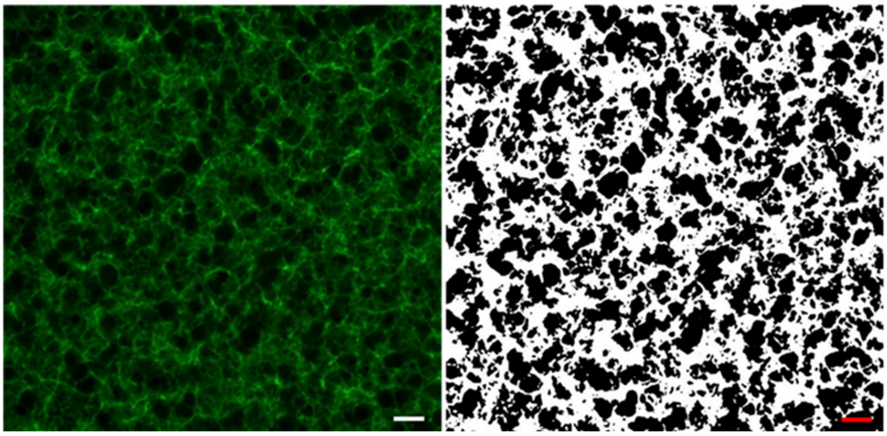

Figure S1. (**A**) ImageJ script applied in batch to convert the fluorescent fibrin images into black and white pixels. (**B**) Representative image of the condition 2.5 mg/mL of fibrinogen and 0.1 U/mL of thrombin with 4% 488-labelled fibrinogen before and after script application on the left and right respectively. The scale bar represents 10 μm.

(A)

```
a = size(StepTime)
b = (a-1000)

lm = sum(LossModulus(b:a,1))/1000;
Sm = sum(StorageModulus(b:a,1))/1000;
T = table (lm, Sm)

 figure ('DefaultAxesFontSize',22)
subplot(1,2,1)
plot (StepTime,LossModulus)
xlabel('Time (s)')
ylabel('Loss Moludus (Pa)')
subplot(1,2,2)
plot (StepTime, StorageModulus)
xlabel('Time (s)')
ylabel('Storage Moludus (Pa)')
```

(B)

```
T =

  1×2 table

     lm        Sm
   ______    ______

   3.0901    88.781
```

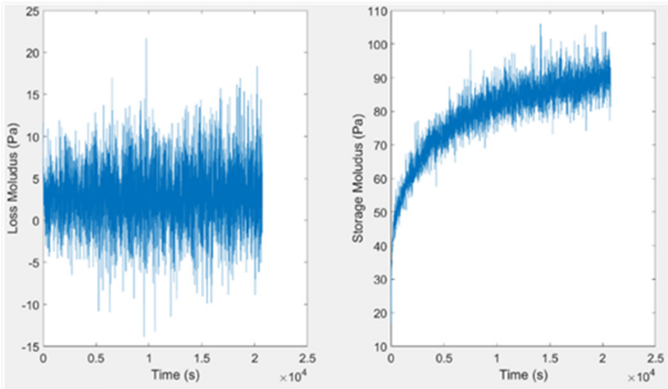

Figure S2. (**A**) MATLAB script applied to the rheometer data. (**B**) Example of MATLAB script output for loss (lm) and storage modulus (sm) for the condition tested with 2.5 mg/mL fibrinogen and 5 U/mL thrombin.

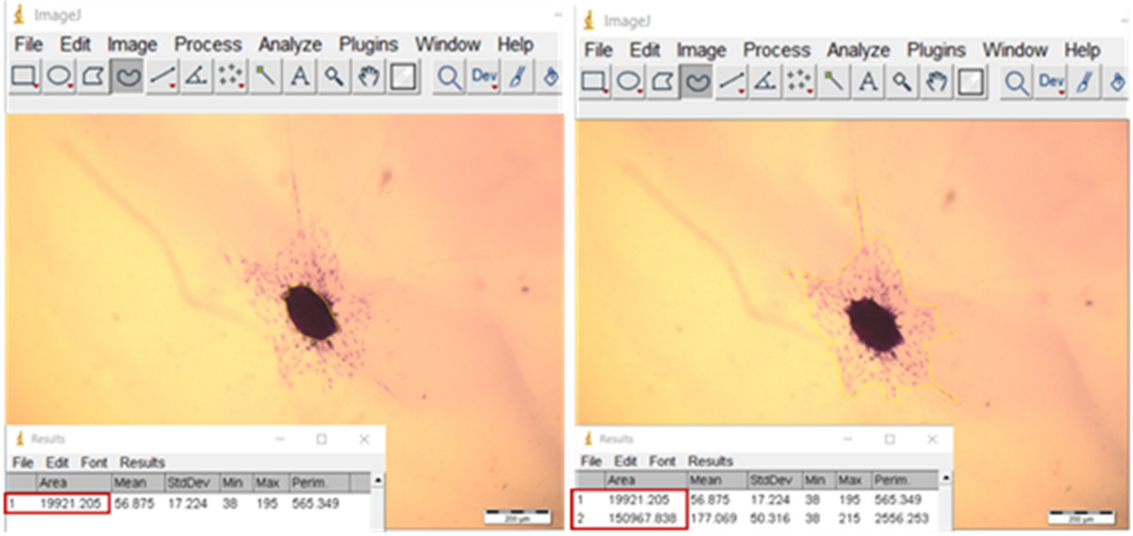

Figure S3. ImageJ analysis of endothelial cell migration area. The left image shows a representative spheroid stained with haematoxylin, with its initial area outlined in yellow. The right image shows the same spheroid, highlighting the total area of cell migration, also outlined in yellow. The condition depicted corresponds to fibrin polymerization at 1.25 mg/ml + 1 U/ml.

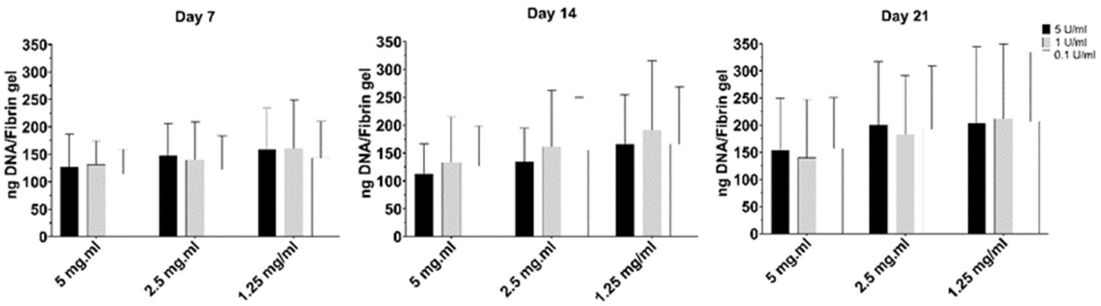

Figure S4. Proliferation of endothelial cells seeded inside fibrin gel over 21 days and regarding the different fibrinogen and thrombin concentrations. The cells were seeded in 100 uL fibrin gel in 96 well plates at 25000 cells/gel. The error bars represent the Standard Deviation. *n* =4.

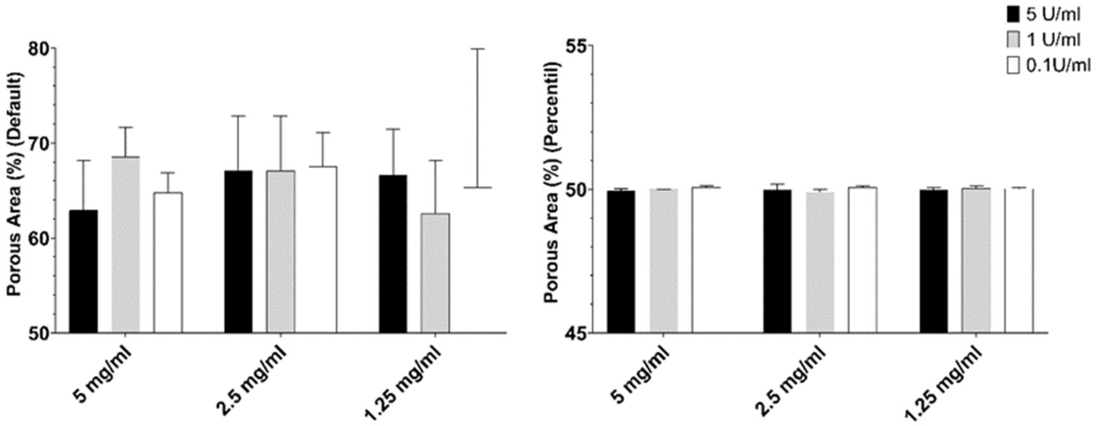

Figure S5. Percentage of area corresponding to pores in the fibrin gel depending on fibrinogen and thrombin concentration. Plot on the left corresponds to the data analyzed with the Default algorithm from ImageJ and plot o the right with the Percentile. The error bars represent the Standard Deviation. *n* =6 to 9.
